# Mechanisms and applications of probiotics in prevention and treatment of swine diseases

**DOI:** 10.1186/s40813-022-00295-6

**Published:** 2023-02-06

**Authors:** Yue Zhang, Yuyu Zhang, Fei Liu, Yanwei Mao, Yimin Zhang, Hao Zeng, Sufang Ren, Lihui Guo, Zhi Chen, Nataliia Hrabchenko, Jiaqiang Wu, Jiang Yu

**Affiliations:** 1 Shandong Key Laboratory of Animal Disease Control and Breeding, Institute of Animal Science and Veterinary Medicine, Shandong Academy of Agricultural Sciences, Jinan, 250100 China; 2 College of Food Science and Engineering, Shandong Agricultural University, Taian, 271018 Shandong China; 3 School of Life Sciences, Shandong Normal University, Jinan, 250014 China

**Keywords:** Probiotic, Swine diseases, Mechanisms of action, Applications

## Abstract

Probiotics can improve animal health by regulating intestinal flora balance, improving the structure of the intestinal mucosa, and enhancing intestinal barrier function. At present, the use of probiotics has been a research hotspot in prevention and treatment of different diseases at home and abroad. This review has summarized the researchers and applications of probiotics in prevention and treatment of swine diseases, and elaborated the relevant mechanisms of probiotics, which aims to provide a reference for probiotics better applications to the prevention and treatment of swine diseases.

## Introduction

Meat is an essential source of nutrients and energy for modern humans due to its balanced chemical nutrition, culinary potential and excellent digestibility. Swine meat has accounted for a large portion of world-wide meat consumption [1]. Pig farming is often accompanied by diseases. Once the epidemic breaks out, it will not only slow down the growth of pigs, reduce the supply of pork [2], but cause huge economic losses for farmers. The outbreak of Africa swine fever since 2018 has brought a devastating blow to the pig industry [3]. Antibiotics have positive effects in the process of swine disease treatment, such as inhibiting or killing pathogenic bacteria, reducing the incidence of disease, regulating the balance of intestinal flora, and promoting growth performance [4]. However, the long-term and extensive use of antibiotics has led to the increase of drug resistance, so the massive use of antibiotics in animal husbandry has attracted great attention among farmers and researchers. The complete ban on the use of antibiotics in animal feed among many countries has led to the growth of research on the use of probiotics to combat bacterial infections in humans and animals. [5]. Probiotics have a significant effect in replacing feed antibiotics and are beneficial to host health when ingested in sufficient quantities, so have been developed as alternative feed additives to the prophylactic use of antibiotics [6]. The review provides an overview on the types of probiotics, the application of probiotics in swine disease treatment and the mechanisms, aiming to provide a reference for the better application of probiotics in swine disease treatment.

## Characteristics of probiotics in pig industry

**Fig. 1:**
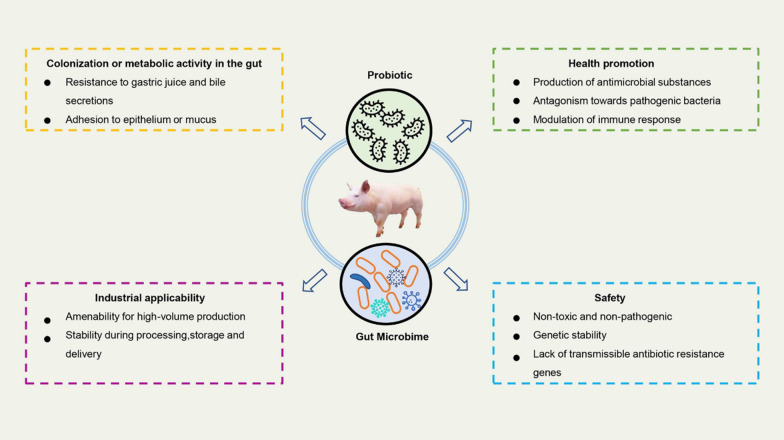
Expected characteristics of probiotics

Probiotics are a class of live microorganisms which contribute to the health of the host. Probiotics are believed to improve the host health through increasing the colonization in host intestine to regulate the balance of the intestinal flora, compete with intestinal pathogenic bacteria, and enhance immunity [7]. The use of probiotics in the swine farming industry must have these following characteristics. Firstly, probiotics survive in the gastrointestinal tract, which means they resist digestion of gastric acid in order to interact with the microorganisms native in the host gut. Secondly, they can improve animal health by stimulating the host’s immune response or through indirect mechanisms, such as reducing damage of pathogenic bacteria. Thirdly, meet production requirements, such as suitability for large-scale production, stable shelf storage, good sensory characteristics, etc. Last but not the least, it has no toxic effects on the host and is not pathogenic [8, 9]. The expected characteristics of probiotics is shown in Fig. 1.

## Most used probiotics bacteria in agricultural breeding

### *Lactobacillus*

*Lactobacillus*, a class of lactic acid-producing Gram-positive bacteria. This genus includes many species such as *Lactobacillus acidophilus*, *Lactobacillus rhamnosus*, *Lactobacillus casei*, *Lactobacillus johnsonii* and so on. *Lactobacillus* promote the intestinal health and growth performance of animals by regulating the expression of cytokines and enhancing intestinal barrier function [10]. Deoxynivalenol (DON) exposure induces liver inflammation and oxidative stress through Keap1-Nrf2 signaling pathway, while *Lactobacillus rhamnosuas* oral administration attenuates the liver dysfunction, improve the antioxidant capacity of liver and protect mice from DON injury [11].

### *Bifidobacterium*

*Bifidobacterium* is a Gram-positive, immobile and strictly anaerobic bacterium. Common probiotic *Bifidobacterium* includes *Bifidobacterium bifidum*, *Bifidobacterium longum, Bifidobacterium breve*, *Bifidobacterium adolescentis*, *Bifidobacterium infantis*, etc. There is large number of *Bifidobacterium* in animal guts, and its state is an indicator to measure the health of the host [12]. Studies have shown that *Bifidobacterium* has abilities to inhibit harmful bacteria, modulate the composition of intestinal microbial, improve gastrointestinal barrier function, and promote the growth performance of animals [13–15].

### *Bacillus*

**Fig. 2:**
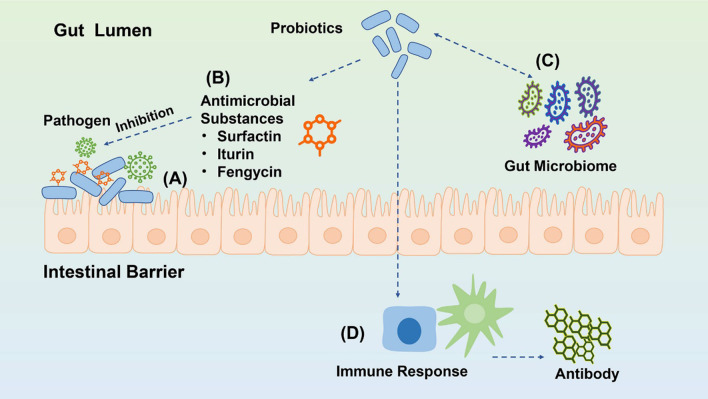
Mechanisms of action of *Bacillus Subtilis*. **A** Competitive adhesion site of pathogenic microorganisms. **B** Production of antimicrobial substances. **C** Regulation of intestinal flora balance. **D** Stimulation of the immune system

*Bacillus* is a Gram-positive, aero-anaerobic or facultative aerobic bacterium. Some bacteria of this genus, such as *Bacillus subtilis* and *Bacillus licheniformis*, are often used as supplement to animal feed and play a vital role. Compared with other non-spore-forming bacteria, *Bacillus* has many advantages because of its endospores. First of all, it can be stored in dry form at room temperature without any impact on its viability. Secondly, the spores are resistant to the threat of extreme environments, which means that *Bacillus* survives in the low pH environment such as gastric acid [16, 17]. Additionally, *Bacillus* secrets enzymes to improve the digestibility of the feed and promote animal growth. When enters the intestine tract, *Bacillus* inhibits the growth of pathogenic microorganisms, and secrete a variety of antibacterial substances to improve the ability of the intestinal immune system, and reduce the occurrence of diseases [18]. Coccidiosis caused by *Eimeria*, which has been recognized as a parasitic disease in chickens that significantly affects the gastrointestinal tract [19, 20]. Dietary supplementation with *Bacillus* strains resisted the negative impacts caused by coccidiosis through different manners [21, 22]. The mechanisms of action of *Bacillus Subtilis* are shown in Fig. 2.

### *Lactic acid bacteria*

*Lactic acid bacteria* refer to a class of non-spore, gram positive bacteria, which is the main product of lactic acid and include multiple species, such as *Lactococci* and *Streptococci* [23]. In animal production, *lactic acid bacteria* are commonly found in fermented feeds. *Lactic acid bacteria* further improve the gastrointestinal function by balancing the intestinal flora, and degrade the macromolecular substances that are not easily absorbed in the body, so they improve the digestive ability of the gastrointestinal tract of animals. The lactic acid, hydrogen peroxide, peptides, etc. produced by *lactic acid bacteria* regulate the immune function and enhance immunity. In addition, *lactic acid bacteria* also reduce the synthesis of cholesterol by reducing the activity of lipid synthetase and assimilating cholesterol. After fermentation, they also synthesize flavor compounds such as acetaldehyde and butanedione to improve the palatability of the feed [24].

### *Yeast*

*Yeast* is a facultative anaerobic eukaryotic single-celled microorganism. In recent years, the interest in using yeasts as probiotics increased, not only for human health but also to improve growth and health of animals and birds, in particular in aquaculture or with respect to industry cattle, pigs, and poultry. *Yeast* cells contains proteins, vitamin B complexes and important trace mineral. And *Yeast* can produce extracellular enzymes, such as amylase, galactosidase, inositol hexaphosphatase, etc., which play an important role in improving the nutritional value and utilization of feed and improving animal production performance [25]. The positive effects of *yeast* on the health and production performance of different animals have been shown in Table 1. Besides improving production performance, *yeast* also regulate balance of intestinal flora, strengthen the immune system, improve animal health, etc.

**Table 1:** The positive influence of yeast supplementation on animal’s health, nutrition and performance [25]

Animal species	Effects
Ruminants	Reduces lactate production or accumulation; Balance ruminal pH; Stimulate the growth of cellulolytic and fibrolytic microorganisms, increasing net fiber intake and digestion; Increasing the total ruminal microflora; Improve the health of the ruminant; Reduce methane emissions
Poultry	Modulate of intestinal microflora; Stimulate immune system; Inhibit the growth of pathogenic bacteria; Increase organic phosphorus utilization; Increasing the egg production and improve the internal egg quality of poultry birds; Improve feed intake; Reduce plasma cholesterol; Promote meat quality of poultry
Pigs	Improve the pig’s gut microflora balance; Proper maturation of the gut associated tissue; Modulate immune response reducing enteric pathogens; Reduce post-weaning diarrhea (PWD) symptoms in pigs; Exhibit growth promoting properties in weaning ang fattening pigs; Improve feed conversion efficiency
Horses	Ameliorates the dysfunction of horse’s ecosystem such as lactic acidosis, colitis, laminitis and enterotoxaemia; Increase in digestibility of diet nutrients; Enhances enzyme production in the hindgut
Aquaculture	Improve growth in juvenile and adult fish; Enhance survival rate; Positively modulate cellular innate immune system parameters; Stimulation of enzymatic antioxidative response of farmed fish

### Other probiotics strains

Some other strains used as probiotics in animal feed are given in Table 2.

**Table 2:** Some other strains used as probiotics in animal feed

Genus	Species	References
*Escherichia*	*Escherichia coli Nissle (EcN)*	[26]
*Enterococcus*	*E. faecalis*	[27, 28]
	*E. faecium*	[29, 30]
	*E. gallinarum*	[31]
	*E. casseliflavus*	[32]
*Aspergillus*	*A. orizae*	[33–35]
	*A. niger*	[36]
*Lactococcus*	*Lc. lactis*	[37]
*Kluyveromyces*	*K. fragilis*	[38]
	*K. marxianus*	[39]
*Leuconostoc*	*L. mesenteroides*	[40]
*Pediococcus*	*P. acidilactici*	[41, 42]
*Streptococcus*	*S. thermophilus*	[43]
	*S. phocae*	[44]

## Applications of probiotics in the prevention and treatment of swine diseases

Some reports have shown that probiotics not only maintain the microbiome balance of intestinal flora, also enhance the immunity and disease resistance of pigs, therefor reducing the severity and risk of disease in animals. In the process of pig framing, the addition of probiotics efficiently reduce the use of antibiotics, so as to improve pig production performance and disease response and other multiple challenges [45]. The application effects of probiotics in pigs are shown in Table 3. This review mainly focuses on the research of probiotics in the prevention and treatment of swine diseases.

### Applications of probiotics in porcine respiratory diseases

Respiratory diseases in pigs are common in modern pork production. And respiratory diseases are often referred to as Porcine Respiratory Disease Syndrome (PRDC) which is caused by the combination of one or more viruses and bacteria [46]. The main pathogens of pigs include viruses such as porcine reproductive and respiratory syndrome virus (PRRSV), swine influenza virus (SIV), pseudorabies virus (PRV), porcine circovirus type 2 (PCV2), porcine epidemic diarrhea virus (PEDV), as well as bacteria such as *Mycoplasma hyopneumoniae*, *Streptococcus*, *Actinobacillus Pleuropneumoniae*, *Haemophilus Parasuis*, and *Bocherichia bronchialsepticae* [47].

The application effect of probiotics in the prevention and treatment of swine respiratory diseases is mainly manifested in improving the immune capacity of pigs. Probiotics (such as *Bacillus subtilis* and *lactic acid bacteria*) play an important role in disease prevention and treatment as live carriers of bacteria. The genome of PCVs encodes two major proteins, replicase (Rep) and capsid protein (Cap). Rep is involved in viral genomic replication, while Cap is the only structural protein of PCVs, and contains multiple antigen epitopes [48]. For example, a recombinant strain expressing the CAP protein of the PCV2 virus was constructed with *Bacillus subtilis*, and it showed that piglets with oral administration of *Bacillus subtilis* had induced a strong humoral immune response by increasing the levels of PCV2-specific immunoglobulin A (IgA) and IgG [49]. Mycoplasma hyopneumoniae (Mhp) is the main pathogen of swine pneumonia, which leads to symptoms such as coughing and wheezing in pigs. Recombinant *Bacillus subtilis* was constructed by using P97 and P46 of *Mycoplasma pneumoniae* as immunogenic proteins, and piglets were immunized by nasal spray using the recombinant strain. The results showed that the secretory immunoglobulin A (sIgA) in nasal swabs and IgG in pig serum had increased significantly after immunization [50]. The level of IgG and IgA antibodies in serum is an important indicator to evaluate the immune capacity. At the same time, sIgA is considered as an immune barrier on the surface of the intestinal mucosa, which prevents pathogens from adhering to intestinal epithelial cells and inhibit the invasion of pathogens [51]. Probiotics as vectors to express specific immunogenic proteins can induce pigs to produce higher levels of antibodies, thereby increasing the immunity of pigs.

In addition, probiotics inhibit the growth and reproduction of pathogens. Studies have shown that co-infected of weaned piglets with *Salmonella cholerae* in pigs and PRRSV leads to *Salmonella* colonization increasing in the lungs and aggravates the symptoms of pneumonia. The results showed that directly dietary supplementation of *Bacillus subtilis* could reduce the *Salmonella* colonization in the lungs and reduce the existence of PRRSV [52].

### Applications of probiotics in porcine digestive tract disease

Digestive tract disease is a common type of pig disease. Although the disease will not cause death of animals, it will affect its growth performance, and then affects the income of farmers. Therefore, many farmers attach great importance to the prevention and treatment of digestive tract diseases. The digestive system is the place where the animal digests and absorbs nutrients. If there are problems with the digestive system of the pig, the digestion and absorption efficiency of pig feed will be reduced, and the weight gain effect of the pig will not be obvious, which will lead to a reduction of farmers’ income. If the digestive system diseases are not treated in time, it is very likely that the gastric mucosa of pigs will be damaged, the immunity will be weakened, and the risk of other diseases in pigs will be increased [53].

Studies have shown that probiotics can reduce the severity of diarrhea and improve the health status of animals. PWD in piglets is associated with enterotoxin-producing Escherichia coli (ETEC) [54]. Dietary supplementation of a variety of probiotics (including *Lactobacillus acidophilus*, *Lactobacillus casei, Bifidobacterium thermophilus*, and *Enterococcus faecalis*) had reduced the severity of diarrhea caused by ETEC F18+ [55]. Oral administration of *Bacillus subtilis* WS-1 at 1.5 × 10^10^ CFU significantly had inhibited diarrhea and mortality in piglets caused by pathogenic *E. coli*, and the study also found that WS-1 also encodes a variety of functional proteins, such as lipopeptides, which may confer antibacterial activity to WS-1 [56]. Newborn piglets in the treatment group with 2 ml of sterile skim milk suspended with viable *Lactobacillus rhamnosus* (5 × 10^8^ CFU/mL) had decreased the diarrhea incidence and increased weaning weight and average daily weight gain [57]. Metabolites of probiotics can also inhibit the replication of pathogens. PEDV is an enteropathogenic virus that causes diarrhea in pigs and is also associated with high morbidity and mortality in piglets. One of the main components of *Lactobacillus plantarum* metabolites is *Lactobacillus plantarum* extracellular polysaccharides (LPE), which elevate the transcription and apoptosis levels of tumor necrosis factor-α (TNF-α), inhibiting the replication of PEDV by regulating the apoptosis mechanism and inducing early apoptosis of damaged cells [58].

In addition, probiotics enhance the intestinal defenses by modulating intestinal flora. The addition of a BLS mixture (a mixture of *Bacillus licheniformis* and *Bacillus subtilis*) to piglet diets increased the relative abundance of *Bacteroides* and *Lactococcus* and decreased the abundance of *Brucella* and *Clostridium* [59]. Studies have shown that *Clostridium* is closely associated with the fermentation of proteins and could increase the risk of diarrhea [60]. In addition, the BLS mixture increased cytokine and TLR-4 levels in the ileum and colon. These findings suggest that BLS mixtures may modulate intestinal flora composition and improve intestinal health.

In addition to being added directly to feed, probiotics are used in feed fermentation as well. Through fermentation technology, anti-nutritional factors such as phytate and soybean antigen protein are decomposed, so as to improve feed digestibility. In addition, fermented feed can also affect the intestinal symbiotic flora, activate immune response, and be beneficial to animal health. For example, feeding wheat fermented by *Lactobacillus reuteri* to weaned piglets could increase the content of short-chain fatty acids and improve the intestinal health of weaned piglets, thereby reducing the incidence of diarrhea in pigs [61]. In addition, studies have shown that fermented soybean meal produced with Lactobacillus and Clostridium butyrate increased the level of immunoglobulin in pig serum and reduced the incidence of diarrhea in weaned piglets [62].

## Mechanisms of action of probiotics

### Regulation of intestinal flora balance

The microbiota in the gastrointestinal tract plays an important role in host metabolism, immunity, digestion, absorption, and development. The gastrointestinal tract is full of microorganisms that are closely associated with the animal itself and the feed it consumes. A good intestinal flora is essential for animal health by ingesting nutrients from the diet and regulates the development and function of the digestive and immune systems to benefit the host.

Probiotics can alter the structure of the intestinal flora. Probiotics improve intestinal health by promoting the growth of beneficial bacteria, inhibiting the multiplication of harmful bacteria, and producing metabolites such as short-chain fatty acids. A balanced chicken GIT is predominated by *Firmicutes*, *Tenericutes*, *Bacteroidetes*, and *Proteobacteria* [65]. Once chickens are infected with *Eimeria*, the balance of intestinal flora is destroyed [63–65]. The use of probiotic modifies gut microbiota composition to benefit the growth of chickens [20, 66]. Study has reported that chickens infected with *Eimeria* significantly reduced the abundance of *Firmicutes* and increased abundances of *Proteobacteria* [22, 64]. The addition of *Bacillus subtilis* improved the adverse impacts of *Eimeria* through enhancing the abundance of *Bacillus*, *Weissella*, *Staphylococcus*, *Bacilli* unclassified and *Turicibacter* [22]. The addition of 500 g/t of *Bacillus subtilis* to weaned piglets’ diets increased microbial β-diversity and relative abundance of *Bacillus*, *Bifidobacterium* and *Clostridium faecium* in the ileum and colon of weaned piglets [67]. *Phylum Firmicutes* and *Bacteroidetes* are dominant bacteria in the porcine intestine [68], *Bacteroidetes enterica* are involved in the degradation of carbohydrates and proteins and play an vital role in maintaining intestinal health through the production of butyrate [69];fermentation of carbohydrates by *Clostridium faecalis* produces butyrate and acetate, along with formate or propionate; *Bifidobacterium* produce acetate to prevent intestinal infections and play an important role in immune regulation [70].

In addition, some substances in probiotics inhibit the growth and reproduction of harmful bacteria to reduce the occurrence of intestinal diseases. For example, the incidence of diarrhea in piglets could be reduced by adding *Saccharomyces cerevisiae* to the diet, which was mainly attributed to the cell wall polysaccharides of *Saccharomyces cerevisiae* [71]. The main active components of yeast cell wall polysaccharides are β-glucan and mannan, which play an important role in improving the immune system and protecting the health of animals [72]. Oligosaccharides promote the formation of beneficial bacteria in the intestine, inhibit the proliferation of pathogenic bacteria and regulate the intestinal flora as a way to maintain intestinal health. Study found that adding 0.3% and 0.5% *Saccharomyces boulardii* yeast wall polysaccharides to the diet could significantly suppressed the growth of *Salmonella* and *Clostridium perfringens* in early weaned lambs [73]. The above findings suggest that probiotics inhibit the growth of harmful bacteria in the intestine tract while increasing the number of dominant bacteria, maintaining the balance of the intestinal flora and thus improving animal health.

### Improvement intestinal mucosal structure and enhancement intestinal barrier function

The structure of the small intestine and the integrity of the intestinal barrier play a pivotal role in the digestion, absorption and transport of nutrients. In general, the digestion and absorption of nutrients in animals is positively correlated with the intestinal villi height and the ratio of intestinal villi height to crypt depth [74]. Studies have suggested that reduced villus height and increased crypt depth may be responsible for nutrient malabsorption, increased gastric secretion, and diarrhea [75]. Yang et al. [76] found that the addition of *Lactobacillus plantarum* during piglet feeding alleviated the decrease in jejunal villi height that occurs after enterotoxigenic *E. coli* attack. *Bacillus subtilis* increase the villi height and the ratio of villi height to crypt depth in the ileum, expanding the absorption area of nutrients and improving digestion and absorption in weaned piglets [67]. In addition, there is a certain relationship between the morphological structure, development and related genes of the intestine. *IGF1*, *IGF-1R*, *GLP-2* and *TGF-β2* are the main markers of intestinal development and cell differentiation [77]. *Bacillus subtilis* upregulated the expression of *IGF1* and *GLP-2* in the jejunal mucosa and yeast hydrolysates upregulated the expression of mRNA of *GLP-2* and *TGF-β2* [78]. Probiotics improve intestinal structure by promoting the expression of genes related to intestinal morphology and intestinal development, helping to increase the total surface area of the intestine, which is an effective guarantee for the absorption of nutrients such as nucleotides [79].

The tight junctions (TJs) between intestinal epithelial cells play an important role in the intestinal mucosal barrier. Damage to TJs leads to increased cell permeability, and bacteria and pathogens in the intestinal lumen penetrate the intestinal mucosa and enter other tissues, organ or circulatory system, thus leading to the occurrence of diseases. Probiotics enhance intestinal TJs and improve the defense of intestinal epithelial cells against pathogenic invasion. Studies have shown that *Lactobacillus plantarum* prevents the adhesion of enterotoxin-producing *E. coli* to intestinal epithelial cells and maintains the integrity of the intestinal barrier [80]. *Bacillus amyloliquefaciens* SC06 (BaSC06) protects the integrity of TJs and villi [81]. The TJs consists of tight junction proteins (claudins) and occludins, and pathogenic bacteria invade the intestines by attacking TJs through various virulence factors. *E. coli* destroys TJs by transferring the occlusion proteins from TJs to the cytoplasm, the absence of which leads to the infection of the organism with pathogenic bacteria [82]. Therefore, tight junction proteins and occlusion proteins play an important role in TJs and intestinal barrier function. *E. coli Nissle* 1917 upregulated the expression of tight junction protein-1 (ZO-1) in intestinal epithelial cells [83]. *Lactobacillus rhamnosus* GG could prevent a decrease in ZO-1 expression induced by *E. coli* O157:H7 [84]. Probiotics increase the trans-epithelial resistance (TER) of intestinal epithelial cells by increasing the expression of occlusion and tight junction proteins, thereby repairing epithelial cell damage caused by pathogenic bacteria.

### Production of antibacterial substances and inhibition the growth of pathogenic microorganisms

Antimicrobial substances, produced by probiotics, such as bacteriocins, hydrogen peroxide, organic acids and biosurfactants, inhibit the growth of pathogenic microorganisms and maintain intestinal health.

Bacteriocins, antimicrobial peptides produced by ribosomes, may inhibit or directly kill pathogenic microorganisms, thereby limiting the colonization ability of pathogenic microorganisms in the intestine. Bacteriocins can induce increased cytoplasmic membrane permeability in bacteria, which leads to cell leakage and inhibition of DNA, RNA synthesis and/or cell wall protein synthesis [85]. For example, nisin from *Streptococcus lactis* acts by forming complexes with cell membrane lipid II precursors, followed by aggregation of polypeptides bound to form discrete pores in the bacterial cell membrane [86].

Another mechanism of action of probiotics in the intestine is to make the intestinal environment unsuitable for the growth of pathogenic microorganisms by lowering pH. *Lactic acid bacteri*a and commensal microbiota ferment carbohydrates in the gastrointestinal tract, leading to the production of metabolites such as acetic acid, formic acid, succinic acid and lactic acid, making the intestinal environment acidic and inhibiting the growth of pathogenic microorganisms. Organic acids (especially lactic and acetic acids) inhibit the growth of many pathogenic bacteria in the gastrointestinal tract [87]. The undissociated form of lactic acid acts as a permeabilizing agent for the outer cell membrane of Gram-negative bacteria, followed by dissociation into the bacterial cytoplasm, exerting a bactericidal effect by accumulating ionized forms of organic acids and other antibacterial compounds within the cytoplasm. Studies have shown that the strong inhibitory effect of *Lactobacillus rhamnosus* GG on *Salmonella typhimurium* is due to the production of lactic acid [88]. Lactic acid affects the expression of *HilA* and *InvF* virulence factors in Salmonella [89].

In addition to the production of organic acids and bacteriocins, the production of hydrogen peroxide by symbiotic microbiota and *lactic acid bacteria* may be an important antimicrobial mechanism. Hydrogen peroxide reduces the virulence of pathogenic microorganisms, decreases the invasion of epithelial cells by pathogenic microorganisms, or causes the death of intestinal pathogenic microorganisms by diffusing within epithelial cells to alter gene transcription and signal transduction [90]. Biosurfactants are a class of compounds with surface and emulsifying activity that cause increased permeability of cells by disrupting or dissolving cell membranes [91]. For example, bacteriocins and biosurfactants produced by *Lactobacillus casei* MRTL3 could inhibit *Listeria monocytogenes*, *Staphylococcus aureus*, *Shigella* and *Pseudomonas aeruginosa* [92].

### Enhancement of immune capacity

Probiotics stimulate immune system of the organism by increasing the production of antibodies and activating immune cells. Vibriosis is a common bacterial disease, which has a negative impact on economically farmed shrimp, marine fish and some freshwater fish [93, 94]. The dietary probiotic can significantly modulate the immune responses to reduce the mortality caused by several *Vibrio* species [95–97]. Serum immunoglobulin is one of the indicators of the immune status of the organism, and the addition of *Bacillus subtilis* and *Bacillus licheniformis* to the diet of weaned piglets could promote the production of serum IgG [98]. Fermented soybean meal produced with *Lactobacillus* and *Clostridium butyrate* enhanced immunity in weaned piglets by increasing serum IgG and IgA levels [62]. sIgA is the main immunoglobulin in the mucosal system and essential to protect the mucosal surface from toxins, viruses and enteric intestinal pathogens. *Lactobacillus* BS15 could increase intestinal sIgA levels and delay the decline in sIgA levels, which contributed to the maintenance of intestinal health [99].

Toll-like receptors (TLRs) are one of the key recognition receptors in the innate immune system and are expressed in various intestinal mucosal cells such as mucosal epithelial cells, macrophages, and dendritic cells. Increased TLR expression results in the release of cytokines including tumor necrosis factor (TNF), interleukin-4 (IL-4), and interferon-γ (IFN-γ) when probiotics are used to stimulate the innate immune system. *Bacillus amyloliquefaciens* SC06 (BaSC06) alleviated intestinal inflammation in fattening pigs by regulating the expression of pro-inflammatory cytokines IL-6, IL-8 and MCP1 in the intestinal mucosa [100]. *Lactobacillus fermentum* and *Pedioccocus acidilactici* reduced the concentration of IL-6, IL-1β, IFN-γ in the serum of weaned piglets, which helped to reduce the damage caused by inflammation [101].

Changes in T cell subsets in the peripheral blood are an important indicator of overall immunity levels, and mature T cells can be divided into two main subpopulations (CD4+ and CD8+) based on the cell surface proteins they express. CD4+ T cells are associated with major histocompatibility complex (MHC) class II molecules and act as helper or inflammatory cells in response to exogenous antigens. CD8+ T cells are associated with MHC class I molecules and play a key role in resistance to endogenous antigens [102, 103]. The ratio of CD4+ and CD8+ T lymphocytes is closely related to the immune function. For example, oral administration of *Bacillus subtilis* and total inactivation of PEDV resulted in an upregulated ratio of CD4 + CD8 + and enhanced proliferation of memory T cells in the intestinal mucosa-associated lymphocytes of pigs [104].

### Inhibition for the colonization of pathogenic microorganisms

Pathogenic microorganisms have binding molecules on their surfaces that can interact with host cell membranes in a similar manner to antigens. Pathogenic microorganisms have binding molecules on their surfaces that can interact with host cell membranes in a similar manner to antigens. For example, the *Lactobacillus plantarum* CCMA 0743 strain was able to reduce the adhesion of *Salmonella* to two types of cells (Caco-2 and HT-29) [105]. *Lactobacillus plantarum* ZLP001 has the ability to inhibit the adhesion of ETEC to IPEC-J2 cells, and the probiotic prevents the adhesion of pathogens via a competitive mechanism at the colonization site [106]. *Lactobacillus reuteri* improves intestinal health by producing extracellular polysaccharides to increase probiotic colonization in the intestine and inhibits the proliferation of enterotoxin-producing *E. coli* [107]. To gain this competitive advantage, probiotics can alter the intestinal environment by creating inhibitory compounds, lowering pH, and competing for nutrients [108].

In addition, probiotics may weaken the flagellar motility of intestinal pathogenic microorganisms, thereby preventing their colonization of the intestinal tract. The flagellum is the motility organ of bacteria and foodborne pathogens (e.g., *Salmonella typhi*) need to actively move the flagellum in order to cross the intestinal epithelial cells. Following the use of the flagellar inhibitor Sal4, *Salmonella typhimurium* was found to remain non-invasive in infected mice [109].

**Table 3:** Application effects of probiotics in pigs

References	Tested animals and adding time	Probiotics and supplemental levels	Effects
Yi et al. [110]	Weaned pigs (21 days of age)	5 × 10^10^CFU/kg *Lactobacillus reuteri* LR1	Improved intestinal morpho-logical structure; Increased intestinal absorption area; Increased in digestibility of diet nutrients; Improved growth performance
He et al. [111]	Weaned pigs (21 days of age)	10 ml 1 × 10^9^CFU/ml *Lactobacillus johnsonii* L531	
Sun et al. [55]	Weaned pigs (21 days of age)	*L. acidophilus*, *L. casei*, *B. thermophilum* and *E. faecium* with the concentration of 0.25 × 10^8^ CFU/g for each strain	
Fu et al. [78]	Weaned pig1s (body weight of 6.89 ± 0.15 kg)	2 × 10^9^CFU/kg *Bacillus coagulans* , or 2.5 × 10^10^CFU/kg yeast hydrolysate	
Inatomi et al. [112]	Pregnant sows	1 × 10^6^CFU/g *B. mesentericus* TO-A、1 × 10^6^CFU/g *C. butyricum* TO-A, 1 × 10^8^CFU/g *E. faecalis* T-110	Improved immune capacity; Reduced the incidence of intestinal diseases such as diarrhea and intestinal inflammation
Long et al. [71]	Weaned pigs (32 days of age)	1 × 10^10^CFU/g yeast	
Li et al. [113]	Weaned pigs (25 ± 2 days of age)	1 × 10^7^CFU/kg *B. subtilis*	
Li et al. [114]	Weaned pigs (21 days of age)	10ml 5 × 10^9^CFU/ml *Lactobacillus delbrueckii*	
Zhao et al. [115]	Weaned pigs (21 ± 2 days of age)	6 × 10^6^CFU/kg *Lactobacillus casei*	
Zhang et al. [116]	Weaned pigs (1 days of age)	10ml 1 × 10^9^CFU/ml *Lactobacillus rhamnosus* GG	Modulated intestinal micro-flora; Enhanced epithelial barrier
Ding et al. [117]	Weaned pigs (21 days of age)	500 g t^− 1^ *B. subtilis* DSM 32,315	
Zhang et al. [118]	Weaned pigs (26 ± 1 days of age)	1 × 10^9^CFU/kg *Clostridium butyrate*	
Cao et al. [100]	Finishing pigs	1 × 10^8^CFU/kg BaSC06	

## Prospect

Due to the increase in antibiotic resistance and the potential harm to human health, many countries have banned the addition of antibiotics to feeds. In the context of the ban on antibiotics in feed, probiotics have become an alternative to antibiotics and are widely used in pig farming industry. Probiotics have the effect of regulating intestinal flora, improving immunity and intestinal mucosal barrier function, inhibiting the growth of pathogenic microorganisms, etc., Therefore, they can improve the immunity of pigs and are beneficial to the prevention and treatment of swine diseases. However, there are some issues regarding the application of probiotics that need to be addressed. Firstly, the effect of probiotics depends on the specific strain, dose and environment, as well as the host specificity, so the results of different studies may vary when evaluating probiotics. Secondly, although the good adhesion of probiotics is beneficial to its action on the intestinal mucosa, it is also possible to increase the movement of bacteria and make animals sick. Thirdly, the possibility of probiotics used in animal feed entering the human food chain cannot be completely excluded. Although probiotics have been used in the pig farming industry for many years, the mechanism of probiotics in the prevention and treatment of swine diseases and the problems existing in the application of probiotics still need to be further investigated in order to play a greater role in the pig farming industry.

## Data Availability

Not Applicable.

## Abbreviations

DON: Deoxynivalenol
PWD: Post-weaning diarrhea
PRRSV: Porcine reproductive and respiratory syndrome virus
SIV: Swine influenza virus
PCV2: Porcine circovirus type 2
PEDV: Porcine epidemic diarrhea virus
IgG: Immunoglobulin G
IgA: Immunoglobulin A
sIgA: Secretory immunoglobulin A
ETEC: Enterotoxin-producing *Escherichia coli*
TJs: Tight junctions
